# A Pilot Study of Morphometric Analysis of Choroidal Vasculature *In Vivo*, Using En Face Optical Coherence Tomography

**DOI:** 10.1371/journal.pone.0048631

**Published:** 2012-11-26

**Authors:** Mahsa Sohrab, Katherine Wu, Amani A. Fawzi

**Affiliations:** Department of Ophthalmology, Northwestern University, Feinberg School of Medicine, Chicago, Illinois, United States of America; Medical University Graz, Austria

## Abstract

**Purpose:**

To study the ability of volumetric spectral domain optical coherence tomography (SD-OCT) to perform quantitative measurement of the choroidal vasculature *in vivo*.

**Methods:**

Choroidal vascular density and vessel size were quantified using en face choroidal scans from various depths below the retinal pigment epithelium (RPE) in 58 eyes of 58 patients with either epiretinal membranes (ERM), early age-related macular degeneration (AMD), or reticular pseudo-drusen (RPD). For each patient, we used the macular volume scan (6×6 mm cube) for vessel quantification, while high-definition (HD) cross-section raster scans were used to qualitatively assess vascularity of the choroidal sub-layers, and measure choroidal thickness.

**Results:**

Of the 58 patients, more were female (66% versus 34% male), of whom 14 (24%) had ERM, 11 (19%) early AMD, and 33 (57%) RPD. Compared to intact choriocapillaris in all ERM (100%), none of the RPD and only 5/11 (45%) early AMD eyes had visible choriocapillaris on either cross section or C-scans (p-value<0.001). When comparing select regions from the most superficial C-scans, early AMD group had lowest vascular density and RPD had highest (p-value 0.04). Qualitative evaluation of C-scans from all three groups revealed a more granular appearance of the choriocapillaris in ERM versus increased stroma and larger vessels in the RPD eyes.

**Conclusions:**

SD-OCT can be used to qualitatively and quantitatively assess choroidal vascularity *in vivo*. Our findings correlate to previously reported histopathologic studies. Lack of choriocapillaris on HD cross-sections or C-scans in all RPD and about half of early AMD eyes suggests earlier choroidal involvement in AMD and specifically, RPD.

## Introduction

The role of choroidal vasculature in the pathogenesis of age-related macular degeneration (AMD) has been explored using various structural and functional approaches [1], [2], [3], [4], [5], [6], [7], [8]. Until recently, studies of the choroid have been limited to postmortem tissue. Histopathologic comparisons of eyes with early AMD to age-matched controls have shown correlations between choriocapillaris loss and drusen density [1], [2], [3], [5], [6], [7], [8]. Choroidal vascular flatmounts in late-stage AMD have shown loss of choriocapillaris underlying retinal pigment epithelium (RPE) atrophy in atrophic AMD with constriction of remaining choriocapillaris and loss of normal choroidal vasculature underlying areas of intact RPE surrounding choroidal neovascular membranes, suggesting diversity of interactions between the choroid and RPE in late AMD [4], [5].

Clinically, indocyanine green angiography has been used to study choroidal vasculature [9], though it does not allow three-dimensional visualization of the choriocapillaris. While many advances have been made in retinal imaging with spectral-domain optical coherence tomography (SD-OCT), visualization of the choroid has remained a challenge. Recently, the sensitivity of choroidal imaging in SD-OCT was improved through enhanced depth imaging [10], [11], [12], [13], [14], [15], [16], revealing that about one-third of patients with advanced AMD have thin choroid significantly below the mean thickness of age-matched controls [10], [11], [12], [13]. Additionally, choroidal thickness was found to be highly correlated with age, axial length, and refraction, emphasizing the importance of controlling for these variables when studying any patient population [16]. More interestingly, choroidal thickness varies on a diurnal basis by as much as 29+/−16 microns in one study, suggesting that it can be an highly variable measure of choroidal vasculature and further emphasizing the need to develop novel approaches to reliably assess choroidal vascular health *in vivo* [16], [17], [18].

Our understanding of the role played by the choroid *in vivo* can be significantly enhanced by detailed choroidal vascular reconstructions. In a previous study, we used multimodal imaging and registered OCT volume scans of reticular pseudodrusen (RPD) lesions and showed the lesions overlapped with the choroidal stroma [19], illustrating that reconstructed high-density volume OCT scans can be used to study the choroidal vascular patterns. In the current study we wanted to explore the use of reconstructed OCT scans to quantify choroidal vasculature in patients with early AMD and various stages of RPD, with the goal of using SD-OCT to obtain quantitative maps of the choroidal vasculature *in vivo*, similar to histopathologic sections and vascular flat-mounts of the choroid. Our results are the first to demonstrate the utility of this approach through comparison to results of prior histopathologic studies of the choroidal vasculature.

## Methods

### Study Population

The study was approved by the Institutional Review Board at the Doheny Eye Institute and adhered to the tenets set forth by the Declaration of Helsinki. A retrospective review of all patients diagnosed with AMD between June 2008 and July 2011 identified 58 patients between the ages of 41 and 97 who had undergone infrared (IR), fundus autofluorescence (FAF) and SD-OCT imaging. Patients scanned with the Cirrus high-definition SD-OCT device (HD-OCT, Carl Zeiss Meditec Inc, Dublin, CA USA) were included. Some patients had red-free (RF) images with the Spectralis HRA+OCT (Heidelberg Engineering Inc, Dossenheim, Germany), though the majority had RF images obtained as part of the standard fluorescein angiography protocol using the Topcon TRC-50IX (Topcon Medical Systems Inc, Paramus, NJ, USA).

The strict criteria for the presence of RPD as described in the Definitions section below was applied to the entire portfolio of imaging studies for each patient. We enrolled patients with epiretinal membranes (ERM) without evidence of AMD to serve as the control group. The eye with the best image quality or most obvious display of the characteristic of interest was selected for each patient in the study. Eyes that were eligible for more than one category (early AMD, RPD, and/or ERM) were excluded. Only one eye per patient was included in the study.

### Image Acquisition

All 58 patients underwent scanning with the Cirrus HD-OCT Model 4000 device, using super-luminescent diode at 840 nm, which achieves 5 microns of axial and 15 microns of transverse tissue resolution. The device captures 27,000 A-scans per second at 2 mm of depth, and the images were viewed with the latest Cirrus HD-OCT software (Version 5.0; Carl Zeiss Meditec Inc, Dublin, CA, USA). As part of the standard Cirrus imaging protocol, all eyes undergo two scanning protocols, a 5-line raster consisting of 4,096 A-scans for each of the 5 B-scans, and a 512×128 Macular Cube volume scan consisting of 128 equally-spaced horizontal B-scans (each composed of 512 A-scans) over a 6 mm square grid. The line scanning laser ophthalmoscope (LSLO) feature also obtained a registered OCT fundus image for each data cube. The Cirrus OCT imaging protocol further requires photographers to repeat OCT volume scans if the summed OCT projection image suggests that significant motion artifact is present.

### Definitions

Identification of patients with evidence of early AMD, RPD, or ERM was based on recognition of characteristic features as seen in the Cirrus OCT and fundus imaging.

**Figure 1:**
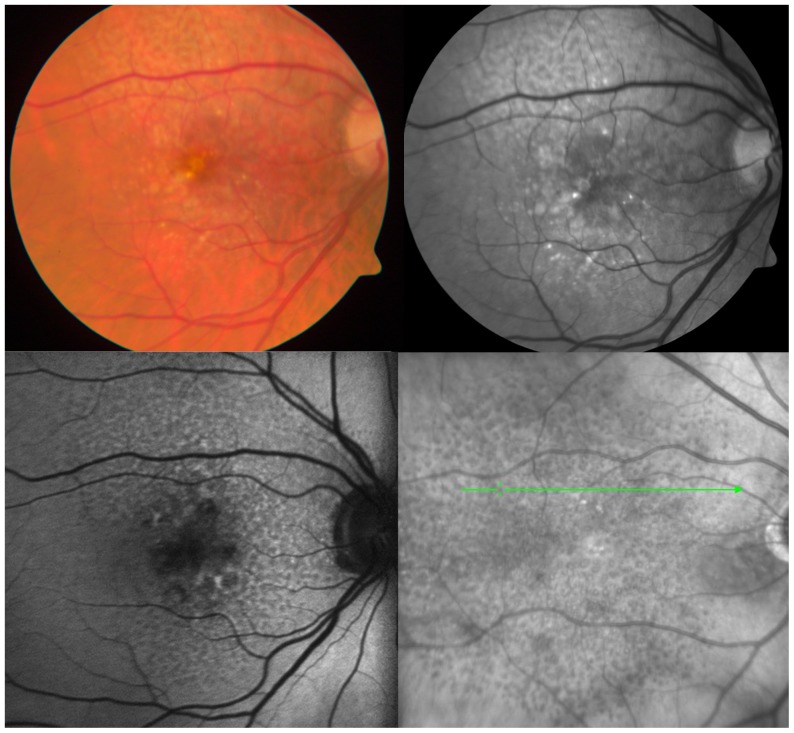
Imaging Characteristics of Reticular Pseudodrusen. Reticular pseudodrusen appear as faint, yellowish, interlacing networks along the arcades on color fundus images (top left) and as light interlacing networks on red-free imaging (top right). Imaging with autofluorescence (bottom left) and infrared (bottom right) improves visualization of reticular pseudodrusen as hypoautofluorescent or hyporeflectant lesions, respectively, extending along the arcades and through the fovea.

Patients with early AMD were identified on the basis of the presence of soft and hard drusen (dry AMD) on imaging. Patients with RPD were identified on the basis of characteristic features on various imaging modalities as defined in previous reports (Figure 1) [19]. Evidence of RPD on RF imaging was defined by the presence of light, interlacing networks ranging from 125 to 250 microns in width. RPD on FAF was defined by the presence of clusters of ill-defined, hypo-autofluorescent lesions interspersed against a background of mildly increased AF occurring in regular and well-defined array. RPD on IR was defined as groups of hyporeflective lesions interspersed against a background of mild hyperreflectance. Advanced RPD was defined as RPD lesions in eyes with evidence of atrophic or neovascular AMD.

### Analysis Protocol

#### En face OCT choroidal sub-layer C-scans

**Figure 2:**
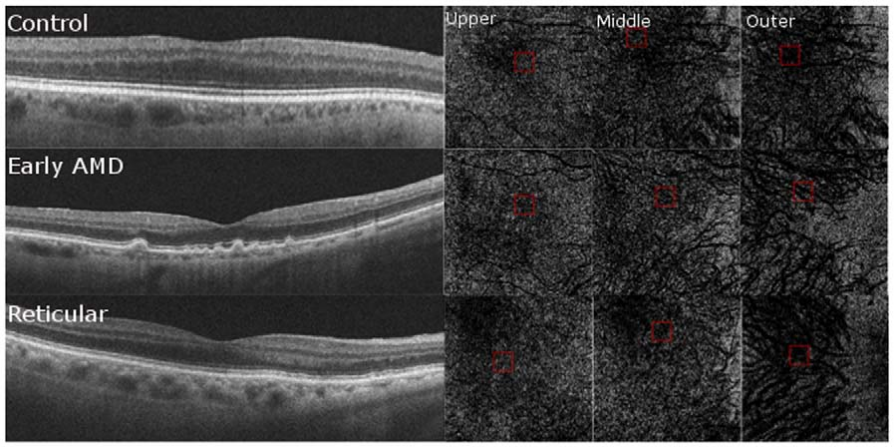
En Face Optical Coherence Tomography (OCT) Choroidal Sub-Layer C-Scans. Examples of 2 micron-thick C-scans obtained from control (top panel), early age-related macular degeneration (AMD, middle panel) and reticular groups (bottom panel). C-scans were obtained from the choriocapillaris or most representative layer below the RPE in the reticular group (center left column), Sattler's or the middle choroidal layer (center right column), and Haller's or the outermost layer (far right column) for further analysis.

The following analysis was performed on each of the 58 patients. OCT volume scans (512×128 macular cubes) obtained on Cirrus HD-OCT were reviewed on the Cirrus version 5.0 software using the advanced visualization feature. As previously described [19], the RPE feature was used to obtain en face slices that were contoured based on each patient's RPE curvature. The slice feature was selected to ensure that the RPE band or sclera were not included in any of the sections. We obtained 2 micron-thick C-scans for each eye at three levels of the choroid, the choriocapillaris, Sattler's layer (middle), and Haller's layer (outermost) to undergo further analysis (Figure 2).

The slice location within each choroidal layer was placed with guidance from the HD OCT scan. In patients in whom choriocapillaris was not visible or difficult to visualize in the en face slice, we confirmed the absence of choriocapillaris using the HD scan and selected the most representative C-scan immediately beneath the RPE.

#### Vessel density on choroidal C-scans (Figure 3)

**Figure 3:**
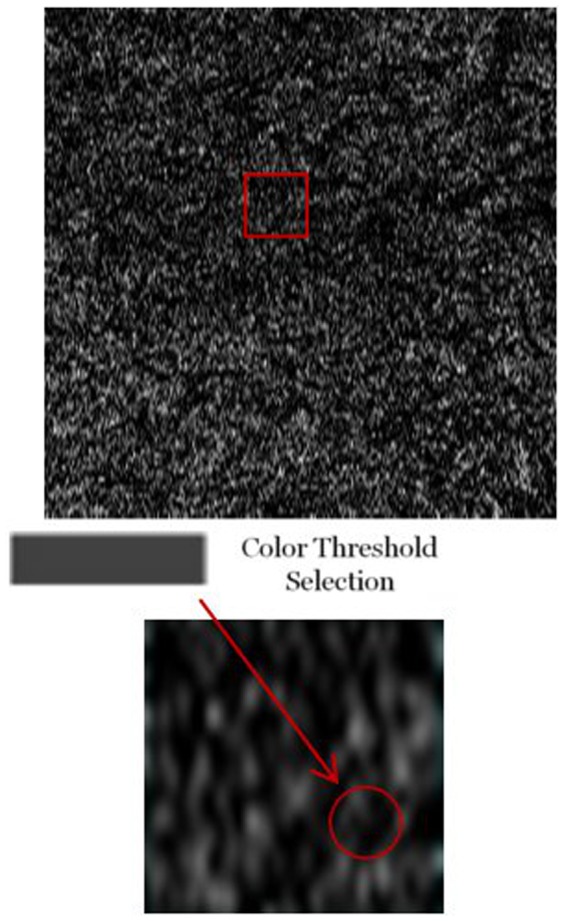
Selecting Pixel Intensity Threshold for Choroidal Vessel Density Analysis. Choroidal C-scans (top image) were obtained using the advanced visualization feature of macular cubes consisting of 512×128 optical coherence tomography volume scans over a 6 mm square grid. Each C-scan was a 2 micron-thick scan from each of the three choroidal layers. A customized image analysis program was used for both full C-scans (top image) and a selected region of each scan (inset) for all patients. A strict threshold of R = 65, G = 65, and B = 65 pixel intensity combination was selected for vessel versus stroma (center).

We developed a custom image analysis program to compare the vessel density present in each choroidal sub-layer for each patient. Since every image is composed of a certain number of pixels, the image analysis program analyses the entire array of pixels, each coded with a specific red, green and blue (RGB) intensity, where the combination of RGB intensities code a specific gray-scale.

**Figure 4:**
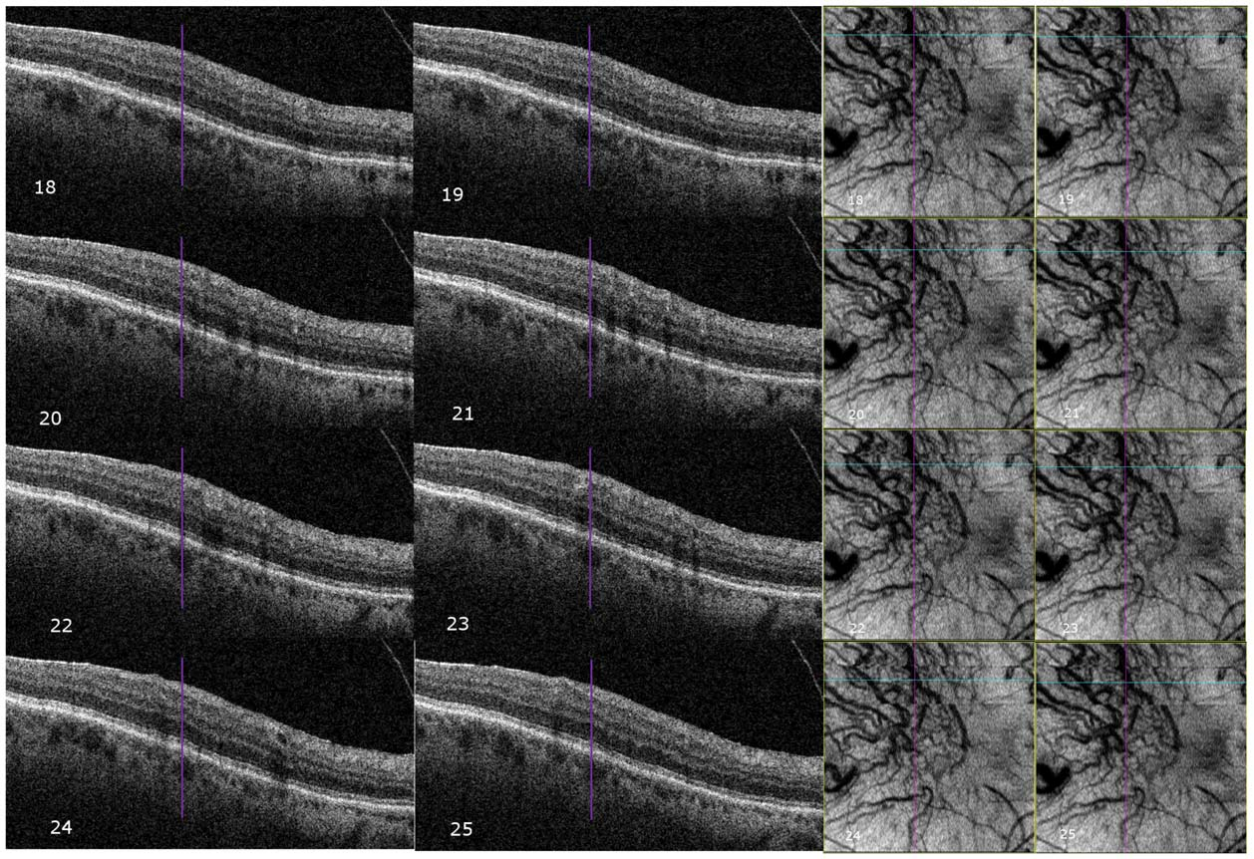
Cross-Correlation of Choroidal Vascular Appearance on Optical Coherence Tomography (OCT) Comparing B- and C-Scans. A large choroidal vessel is identified as a well-defined round hypo-reflective structure in cross-section on individual B-Scans (purple line). Sequential B-scan slices (18–25) are shown with the purple line centered on the same choroidal vessel. On the C-scans shown, the blue horizontal lines correspond to the location of the respective B-scan, while the purple line follows the vertical axis of the corresponding choroidal vessel on C-scan.

Each pixel intensity ranges from 0 to 255, and pixels that are a similar shade of black or white, have the same R, G, and B values. For example, the combination for pure white is R = 255, G = 255, B = 255 while the combination for pure black is R = 0, G = 0, B = 0. We selected a threshold RGB combination from the spectrum of black to white and all pixel combinations with RGB values at or below this threshold were classified as vessel (black) while those above the threshold were considered stroma. In order to confirm that the round hypo-reflective structures seen on individual B-scans correspond to the choroidal vessel lumen, we tracked one of these structures in eight consecutive raster B-scans and cofirmed that the circular lumen coincided with the course of the corresponding choroidal vessel seen on the en face OCT slice (Figure 4). We ran two analyses for all the images, one with a loose cutoff at a gray shade of R = 110, G = 110, B = 110 and one with a stricter cutoff at an almost black shade of R = 65, G = 65, B = 65, selecting the latter cutoff for the vascular analyses of the entire database.

In calculating vessel density, we initially analyzed the full 6×6 mm en-face scans for each patient, which provided an averaged vascular density for the entire macula. We then manually selected representative areas (0.5×0.5 mm) within each en face slice, where the choroidal vasculature was visible to calculate select area vessel density.

#### Measurement of Vessel Diameter in Choroidal C-scans

The customized image analysis tool allowed us to measure the diameter of the individual vessels in each choroidal slice by calculating the distance between the coordinates of the edges of the vessels. Using Cartesian mathematics with the horizontal base as the x-axis and the vertical edge as the y axis, and selecting two coordinates directly across from each other on the border in the x-axis (horizontal dimension), the software calculated the horizontal diameter of the vessels in pixels.

Since some of the vessels in the choriocapillaris were extremely narrow, it was difficult to accurately pinpoint the coordinates of the edges of the vessels. To eliminate error, we programmed the image analysis software to magnify the images from its original size of 644 by 644 pixels to approximately 760 by 760 pixels so we could more easily select the coordinates located on the diameter of the vessels. This discrepancy created by magnification was rectified by modifying the image analysis software to directly proportion the size of the magnified image to the size of its corresponding original image and subsequently correlate the coordinates on the magnified image with the actual coordinates in the original image. Using these adjusted coordinates, the program calculated the diameter of vessels in pixels.

Each C-scan image contained numerous vessels with varying diameters, so to obtain the measurements for the average vessel sizes we took the average of two measurements from the diameter of the largest and smallest visible vessels per image. For the most superficial choroidal layer in eyes with RPD (without visible choriocapillaris), the values were obtained from 5 averaged measurements of the large, medium, and small vessels.

#### Choroidal Thickness

The linear measurement tool was used to measure the choroidal thickness from the base of the RPE to the junction of the sclera and the choroid on the foveal HD raster scans (6 mm lines of 4096 A-scans) obtained on the Cirrus HD-OCT device.

### Statistical Analysis

The analysis of variance (ANOVA) test was used to compare parameters including age, gender and choroidal thickness as well as vessel density (in percentage) and vessel diameter (pixels) for each layer. Analysis of covariance (ANCOVA) was performed for choroidal thickness, vessel diameter and vessel density after adjusting for choroidal thickness and for age and gender. When the ANOVA and ANCOVA were statistically significant (p<0.05), Bonferroni adjusted p-values were calculated for the comparisons.

## Results

### Study Population (Table 1)

**Table 1:** Patient Demographics.

	Control n = 14	Early AMD n = 11	Reticular n = 23	Advanced Reticular n = 10	Overall n = 58	p-value
**Gender**						
***Female***	7 (50%)	4 (36.4%)	19 (82.6%)	8 (80%)	38 (66%)	0.02
***Male, n(%)***	7 (50%)	7 (63.6%)	4 (17.4%)	2 (20%)	20 (34%)	0.02
**Mean Age (+/− SD)**	61(10.7)	76.7(9)	82.6(7)	84.3(7.6)	76.6	<0.001*

AMD = age-related macular degeneration; Control = epiretinal membranes; early AMD = drusen, advanced reticular patients = reticular pseudodrusen with advanced atrophic or neovascular AMD.

* Age Comparisons were statistically significant (p<0.001) between each of the AMD subgroups and control, but not significant between the individual AMD groups.

Of the 58 patients selected for inclusion in the study, there was a twofold preponderance of females, higher in the reticular groups where the ratio of female to male was 4∶1 (p = 0.02). Of all eyes examined, 14 (24%) were classified as controls, 11 (19%) as early AMD, and 33 (57%) as RPD, of whom 10/33 (30%) were classified as advanced RPD (defined in the methods). The control group had the lowest average age while the reticular groups had the highest (p<0.001).

### Qualitative Choriocapillaris Assessment on HD-OCT B-scans and Choroidal slice C-scans (Figures 5, 6, 7, 8)

**Figure 5:**
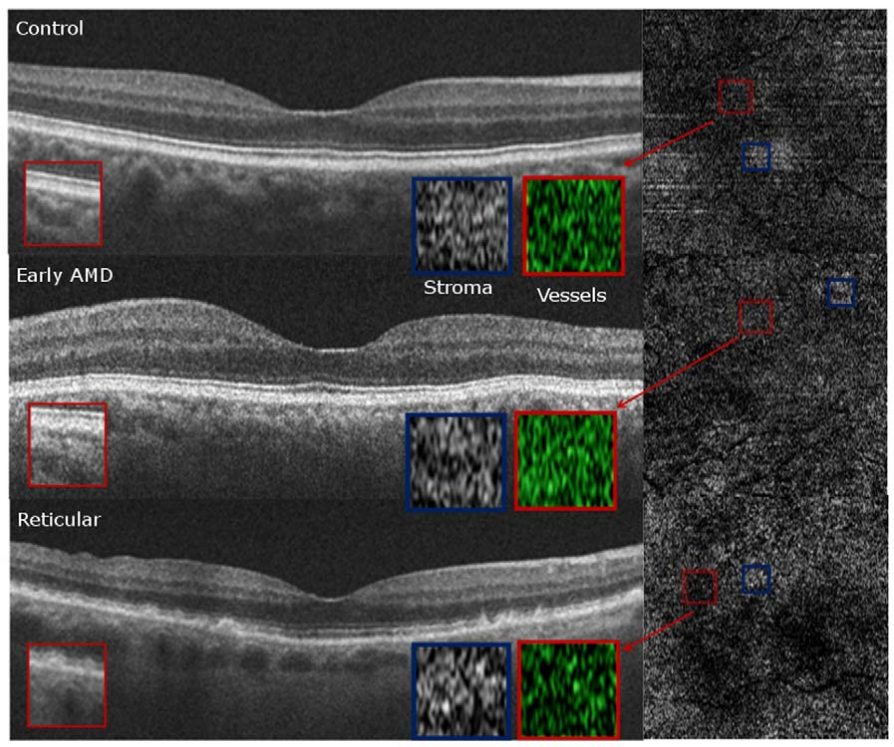
Qualitative Choriocapillaris Assessment Comparing C-Scans and B-Scans (Example 1). When comparing control (top panel), early age-related macular degeneration (AMD, middle panel) and reticular (bottom panel) patients, the appearance of representative C-scans obtained just below the retinal pigment epithelium in reticular groups (bottom right) was qualitatively different from the choriocapillaris scans obtained in the control and AMD groups (top right and center right, respectively). Red boxes on the B-Scans (left) show magnified and colorized selected areas of choroidal vasculature from the regions contained by the smaller red boxes on the C-Scans (right) for each group. Blue boxes on the B-Scans (left) show magnified areas of selected stroma from the regions contained by the smaller blue boxes on the C-Scans (right). Stromal sections demonstrate more patchy whitish regions in the RPD group (bottom panel) as compared to the control and AMD groups (top and middle panels) but similar vessel density due to the presence of larger vessels.

**Figure 6:**
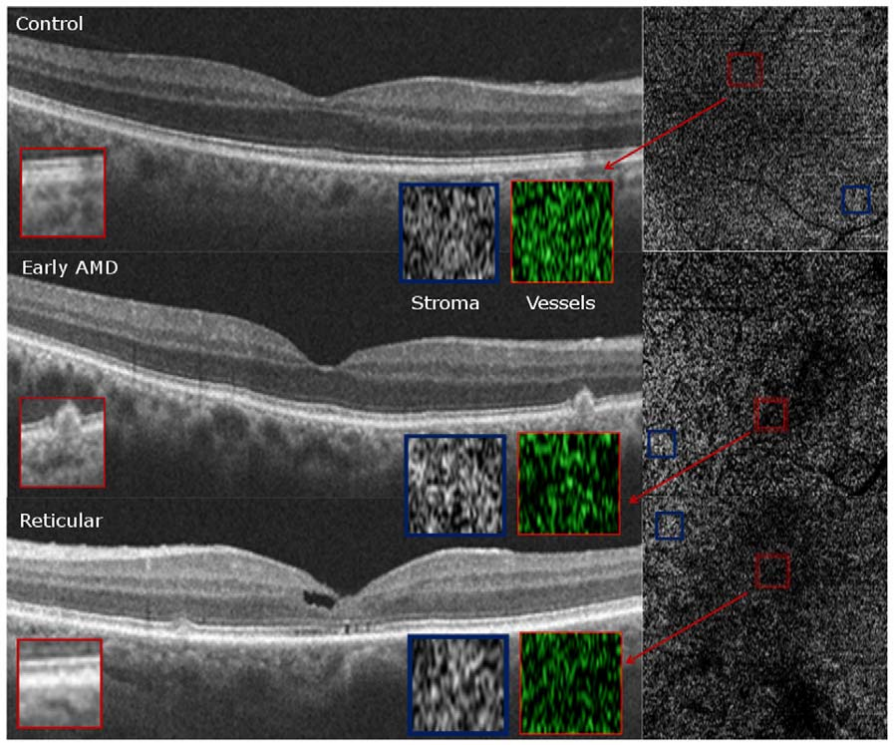
Qualitative Choriocapillaris Assessment Comparing C-Scans and B-Scans (Example 2). Comparing another example of control (top panel), early age-related macular degeneration (AMD, middle panel) and reticular (bottom panel) patients, reveals qualitatively different appearance of representative C-scans obtained just below the retinal pigment epithelium in reticular groups (bottom right) than in the control and AMD groups (top right and center right, respectively). Red boxes on the B-Scans (left) show magnified and colorized selected areas of choroidal vasculature from the regions contained by the smaller red boxes on the C-Scans (right) for each group. Blue boxes on the B-Scans (left) show magnified areas of selected stroma from the regions contained by the smaller blue boxes on the C-Scans (right). Stromal sections demonstrate more patchy whitish regions in the RPD group (bottom panel) as compared to the control and AMD groups (top and middle panels) but similar vessel density due to the presence of larger vessels.

**Figure 7:**
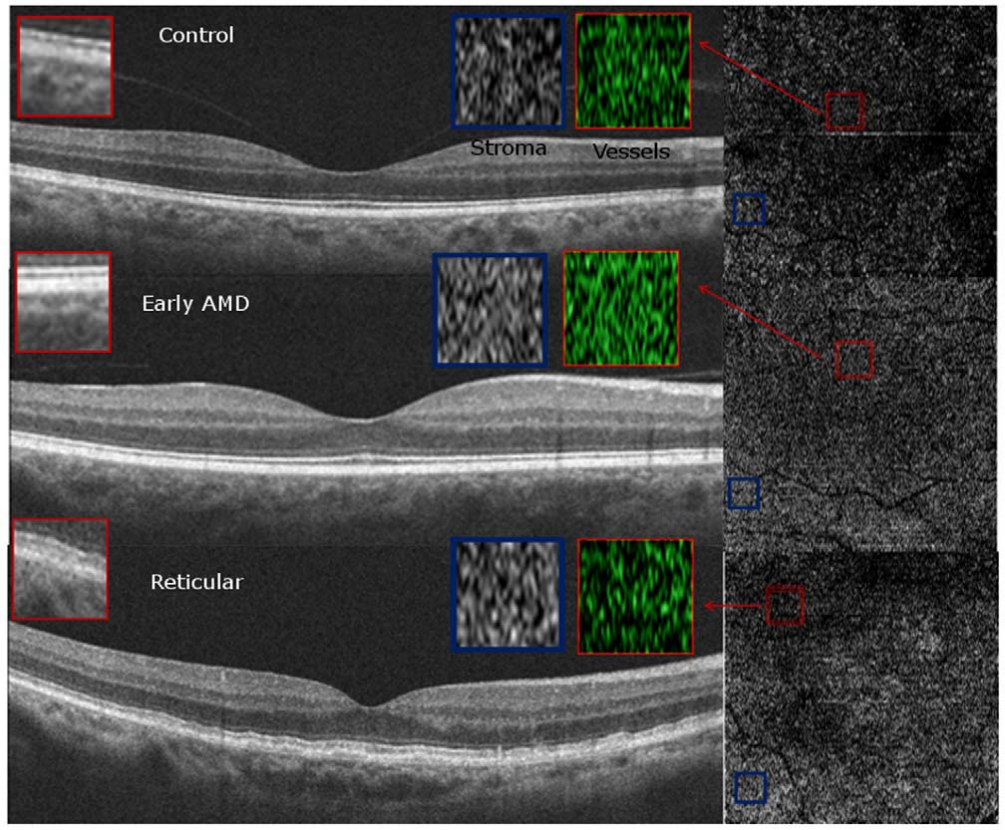
Qualitative Choriocapillaris Assessment Comparing C-Scans and B-Scans (Example 3). Comparisons of control (top panel), early age-related macular degeneration (AMD, middle panel) and reticular (bottom panel) patients reveals qualitatively different appearance of representative C-scans obtained just below the retinal pigment epithelium in reticular groups (bottom right) than in the control and AMD groups (top right and center right, respectively). Red boxes on the B-Scans (left) show magnified and colorized selected areas of choroidal vasculature from the regions contained by the smaller red boxes on the C-Scans (right) for each group. Blue boxes on the B-Scans (left) show magnified areas of selected stroma from the regions contained by the smaller blue boxes on the C-Scans (right). Stromal sections demonstrate more patchy whitish regions in the RPD group (bottom panel) as compared to the control and AMD groups (top and middle panels) but similar vessel density due to the presence of larger vessels.

**Figure 8:**
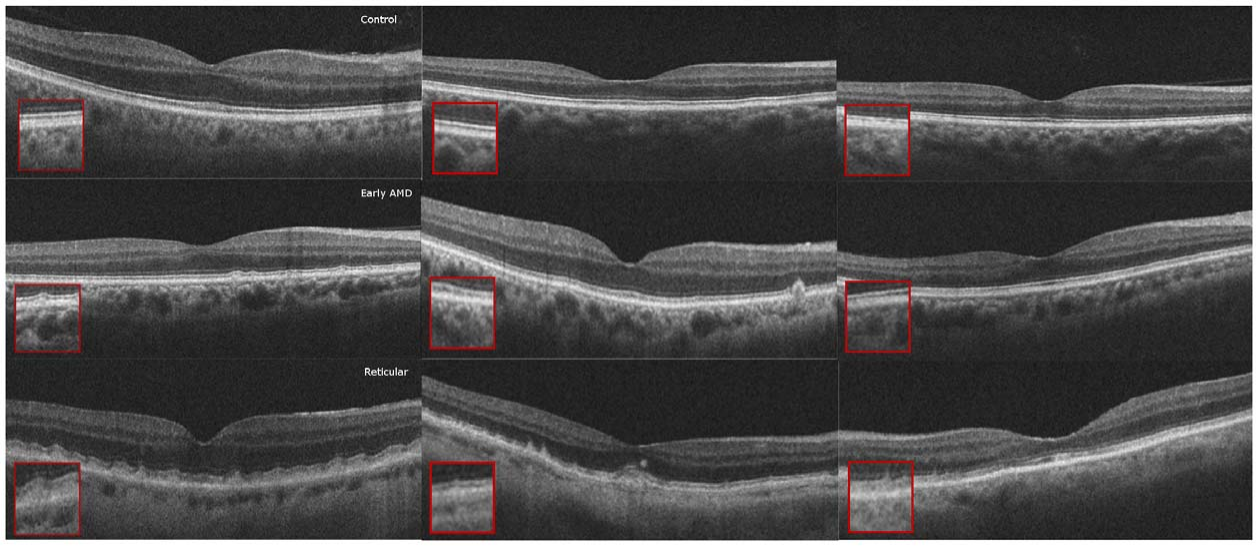
Qualitative High Density (HD) Raster Scan Assessment of Choriocapillaris. HD-raster scans of the control group (top) had a distinct granular-appearing choriocapillaris layer, in contrast to only 45% of the early age-related macular degeneration group (center) and none of the reticular group (bottom), in whom this layer was lacking.

None of the RPD eyes and only 5 (45%) of the early AMD eyes had visible choriocapillaris in the HD-OCT scans, compared to all control eyes (p<0.001, Fisher's exact test).

C-scans of the choriocapillaris layer in control eyes showed a regular, honeycomb-like pattern of alternating vessels and stroma that was distinguishable from the deeper choroidal layers which showed well-defined interlacing vascular channels separated by stromal regions in the middle and outer layers. In contrast, C-scans taken just beneath the RPE in patients without evident choriocapillaris (100% of RPD eyes and 55% of early AMD eyes), showed larger sized vascular channels more closely resembling the middle layer of control eyes (Figures 5, 6, 7). Given the qualitative differences noted on comparing sections from different groups, it is unlikely that the layer immediately external to the RPE in the early AMD and reticular eyes without visible choriocapillaris is analogous to the normal choriocapillaris seen in controls.

### Choroidal vessel density on C-scans (Tables 2 and 3)

**Table 2:** Choroidal Vascular Density on C-Scans: Macular (6×6 mm) versus Regional (500×500 microns).

	Control n = 14	Early AMD n = 11	Reticular n = 23	Advanced Reticular n = 10	p-value^Ac^
	**6×6 mm C-scan Vascular Density (%)**				
***Inner***					
*Model 1*	75.4±2.3	71.7±3.6	74.5±1.9	74.4±2.8	0.86
*Model 2*	78.5±3.2	72.7±4.0	72.1±2.5	72.7±3.5	0.53
***Middle***					
*Model 1*	83.2±1.4	80.1±1.6	80.4±1.1	84.2±2.1	0.18
*Model 2*	83.3±1.9	80.1±1.7	80.5±1.4	84.4±2.3	0.22
***Outer***					
*Model 1*	86.4±1.5	86.8±1.6	86.4±1.1	86.5±1.6	1.00
*Model 2*	85.5±2.0	87.3±1.6	86.1±1.3	86.9±1.9	0.89
	**500×500 micron Regional Vascular Density (%)**				
***Inner***					
*Model 1*	85.2±1.8	79.5±2.8	91.6±1.5	86.5±2.2	0.003*
*Model 2*	86.7±2.5	79.7±3.1	90.7±1.9	86.4±2.8	0.04∧
***Middle***					
*Model 1*	89.6±1.4	90.5±1.5	91.3±1.1	90.3±2.0	0.81
*Model 2*	87.8±1.8	90.3±1.5	92.2±1.3	91.7±2.2	0.36
***Outer***					
*Model 1*	95.7±0.9	95.6±1.0	95.6±0.7	97.2±1.0	0.59
*Model 2*	94.8±1.2	95.7±0.9	95.7±0.8	97.4±1.1	0.41

AMD = age-related macular degeneration; Control = epiretinal membranes; early AMD patients = drusen, advanced reticular patients = reticular pseudodrusen with advanced atrophic or neovascular AMD.

Ac = Analysis of covariance (ANCOVA) p-value. Model 1. Adjusting for choroidal thickness. Model 2. Adjusting for age and gender. When the ANCOVA was statistically significant (p<0.05), Bonferroni adjusted p-values were calculated for the pairwise comparisons. NS = non-significant (p>0.05).

* When comparing Drusen vs early reticulars, p = 0.004, while all other comparisons were non-significant.

∧ When comparing Drusen vs early reticulars, p = 0.04, while all other comparisons were non-significant.

**Table 3:** Choroidal Vessel Density by Choroidal Sublayer.

Vessel Density (%)	Control	Early AMD	Reticular
***Inner***	63.8%–91.2% (76.5%)	67.2%–78.7% (72.2%)	54.8%–91.0% (73.8%)
***Middle***	75.9%–88.4% (83.6%)	74.9%–84.5% (80.3%)	69.7%–89.9% (80.%)
***Outer***	79.3%–93.9% (87.2%)	72.4%–94.4% (87.2%)	76.9%–91.6% (85.9%)

Full slices (6×6 mm) data; Control = epiretinal membranes; early AMD patients = drusen, reticular patients = reticular pseudodrusen with or without advanced atrophic or neovascular AMD, average densities in parentheses.

In order to understand the relationship between choroidal thickness and vascular density, we explored the average vessel density in each full 6×6 mm choroidal slice as a function of choroidal thickness. The deepest choroidal layers had the highest vascular density, regardless of the range of choroidal thickness (Graph S1). Overall, eyes with thicker choroids were found to have higher vascular densities in each choroidal sublayer (Graph S1). Based on this finding, we decided to control for choroidal thickness when evaluating vascular densities.

In Table 2 we tested two models of analyzing the data for covariance, with the first adjusting for choroidal thickness and the second adjusting for age and gender. Comparing the choroidal vascular density between groups using an entire 6×6 mm slice (full scan) versus a smaller selected region (500×500 micons) for each choroidal sublayer, we did not find statistically significant sublayer vessel density differences in the full slice group between groups but did find statistically significant differences in the smaller selected regions, with the lowest vascular density in the early AMD group and the highest density in the reticular group (p-value = 0.0003). Controlling for age and gender, superficial choroidal thickness in the small region remained statistically significant (p = 0.04). Detailed raw values of vessel density data in each group are shown in Table 3.

### Measurement of Vessel Diameter in choroidal C-scans (Table 4)

**Table 4:** Choroidal Vessel Diameter.

	Control n = 14	Early AMD n = 11	Reticular n = 23	Advanced Reticular n = 10	p-value	Early AMD vs Reticular
**Diameter (pixels)**						
***Inner***						
*Crude*	4.43±0.98	4.18±0.36	–	–	0.60	
*Adjusted*	4.49±0.38	4.19±0.45	–	–	0.54	
***Middle***						
*Crude*	8.31±1.22	7.51±2.22	7.36±2.47	7.52±1.47	0.61^A^	0.87^A^
*Adjusted*	8.30±0.78	7.51±0.66	7.36±0.51	7.52±0.93	0.83^Ac^	0.87^Ac^
***Outer***						
*Crude*	19.49±0.07	18.38±2.07	17.61±4.22	18.02±3.21	0.61^A^	0.26^A^
*Adjusted*	19.45±1.16	17.36±1.44	17.98±0.88	18.51±1.30	0.63^Ac^	0.84^Ac^

AMD = age-related macular degeneration; crude versus adjusted for age and gender; Control = epiretinal membranes; early AMD patients = drusen, advanced reticular patients = reticular pseudodrusen with advanced atrophic or neovascular AMD.

A = Analysis of variance (ANOVA) p-value.

Ac = Analysis of covariance (ANCOVA) p-value, adjusting for age and gender. When the ANOVA or ANCOVA was statistically significant (p<0.05), Bonferroni adjusted p-values were calculated for the pairwise comparisons.

Choriocapillaris vessel diameter measured in the horizontal axis in controls ranged from 2.8 to 6.5 pixels (average 4.42), middle choroidal vessels 7.0 to 10.8 pixels (average 8.31), and deeper choroidal vessels 15.6 to 22.1 pixels (average 18.38). Vessel diameters were similar in the early AMD and RPD groups (in layers in which they were present), without statistically significant differences. Given the cube is 6×6 mm, with 512× 128 A-scans, the pixel separation in the horizontal direction is 6000/512 or 11.7 microns (and 6000/128 in the vertical direction or 47 microns), hence the choriocapillaris horizontal dimensions were on average 50 microns, the middle vessels ∼100 microns, and the large vessels ∼215 microns.

### Choroidal Thickness (Table 5)

**Table 5:** Choroidal Thickness.

	Control n = 14	Early AMD n = 11	Reticular n = 23	Advanced Reticular n = 10	p-value
**Thickness (Average +/− SD, microns)**					
***Crude***	207.0+/−42.5	228.6+/−58.4	163.4+/−68.5	179.0+/−99.4	0.04*
***Adjusted***	209.7+/−20.6	198.1+/−24.7	169.4+/−16.7	188.8+/−23.6	0.50^Ac^

AMD = age-related macular degeneration; crude versus adjusted for age and gender; Control = epiretinal membranes; early AMD patients = drusen, advanced reticular patients = reticular pseudodrusen with advanced atrophic or neovascular lesions.

* Analysis of variance (ANOVA) p-values. Pairwise comparisons were statistically significant between Drusen and early reticulars (p = 0.04), but not significant when comparing other subtypes of AMD (p>0.05).

Ac : Analysis of covariance (ANCOVA) p-value, adjusting for age and gender. When the ANOVA or ANCOVA were statistically significant (p<0.05), Bonferroni adjusted p-values were calculated for the pairwise comparisons.

Overall, choroid was thicker in the control and early AMD groups and thinner in the RPD and advanced RPD groups (p = 0.04), though the differences were not statistically significant after controlling for age and gender (p = 0.5). We examined the effect of age and gender on choroidal thickness, and found a trend for females to have thinner choroids up to age 80, after which there was reversal of the pattern, though differences were not statistically significant (Graph S2).

## Discussion

To our knowledge, this is the first study to evaluate choroidal vascular patterns and density using reconstructed SD-OCT volume imaging. While histopathologic studies have documented a range of choroidal vascular changes in normal aging and in AMD [1], [2], [3], [4], [5], [6], [7], [8], the exact sequence of events remains a subject of considerable debate. The ability to perform quantitative choroidal vascular analysis *in viv*o as shown in our study opens avenues for longitudinal detailed study of the choroid in aging eyes and an opportunity to address these controversies definitively.

**Figure 9:**
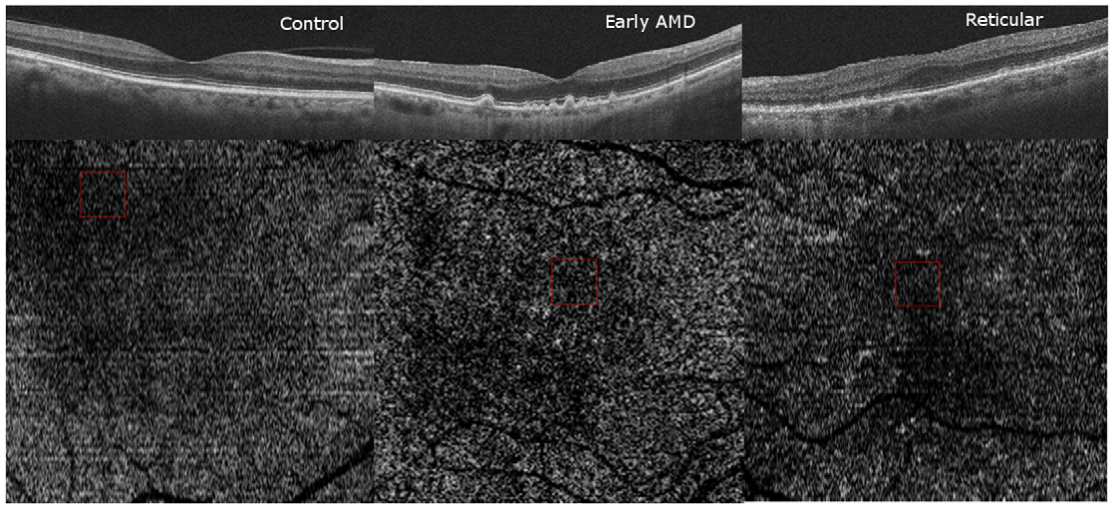
Altered Curvature of the Retinal Pigment Epithelium (RPE). Analyzing 6×6 mm C- scans obtained in the most superficial choroidal layers for all groups were affected by the variance in curvature of the RPE, especially in eyes with thin choroid (middle panel). Examples of high density (HD) raster scans and 6×6 mm C-scans from the control (left), early age-related macular degeneration (AMD, center) and reticular (right) groups are shown. In order to overcome these differences, selected 1×1 mm representative areas from the full C-scans were used for further analysis (red squares, bottom panel).

In evaluating choroidal vessel density, we utilized two different approaches. Vessel density in the 6×6 mm *en-face* C-scan provided an averaged macular vascular density and found similar densities among the different groups. We found that approach suffered from segmentation artifact, especially when evaluating the choriocapillaris, which might be related to RPE curvature especially in eyes with thin choroid (Figure 9). Qualitative evaluation of the entire C-scans in these eyes showed non-uniform appearance of the vascular patterns suggesting patchy vascular loss (Figures 5, 6, 7). We therefore performed an additional quantitative analysis on manually selected regions (500×500 microns) with visible vasculature (Figures 5, 6, 7). Using these select areas, we found that early RPD eyes had statistically significant increased superficial choroidal vascular density compared to early AMD, after adjusting for age and gender (p = 0.04) and choroidal thickness (p = 0.004). Taken together with absent choriocapillaris on HD-OCT cross sections in 100% of RPD eyes, we believe that the most superficial C-scan analyzed in these eyes included larger choroidal vessels interspersed with atrophic choriocapillaris. It is further possible that residual “ghost” or non-perfused choriocapillaris, which are likely less than a horizontal pixel (11 microns), are under-estimated by this approach due to lack of adequate resolution.

Qualitatively, the regular “honeycomb” pattern of the most superficial C-scan in control eyes was distinctly absent in reticular eyes, further validating our conclusions (Figures 5, 6, 7, 8). Instead, RPD eyes show interlacing larger diameter choroidal vessels in the most superficial vascular layer of the choroid (Figures 5, 6, 7, 8). Qualitative evaluation of HD-raster B-scans further confirmed the lack of a “granular appearing” choriocapillaris in the immediate sub-RPE region of RPD eyes (Figure 8). The lack of statistically significant differences in superficial vascular density of the full slices reflects a combination of the patchy distribution of the larger vasculature and dense stroma noted on C-scans in RPD eyes. In contrast, analysis of selected areas with visible vascular patterns demonstrated focally increased vascular density in RPD.

HD cross-section OCT scans showed that less than half of eyes with early AMD had visible choriocapillaris. This is similar to histopathologic findings of choriocapillaris atrophy and vascular dropout in areas of basal laminar and basal linear deposits [1], [2], [4]. Furthermore, we found statistically significantly decreased choroidal vessel density in the superficial choroid in early AMD eyes versus controls (79.7% versus 86.7%, p = 0.04). Choriocapillaris diameter in early AMD eyes was slightly reduced compared to control eyes, though this was not statistically significant (4.19 versus 4.49 pixels, respectively). In control eyes, we found an overall choriocapillaris vessel density (6×6 mm) of 78.5%, closely paralleling 79.6% reported in histopathologic studies, supporting the utility of OCT scans as a reliable tool for quantitative choroidal vascular mapping *in vivo*.

The vessel diameters found in this study are consistent among eyes, averaging 40–50 microns for the choriocapillaris compared to 100 and 200 microns for medium and large choroidal vessels, respectively. These measurements are consistent with histopathologic evidence in the posterior pole, though our approach probably leads to overestimation of the choriocapillaris size. The average macular choriocapillaris size on histopathology is ∼20 microns as compared to the lateral resolution of the OCT system, which is 15 microns, making it inadequate for accurate choriocapillaris size assessment [20]. This problem could potentially be solved by incorporating the axial dimension of the choriocapillaris (from cross-sectional b-scans) into the calculation of choriocapilaris diameter. By assuming the choriocapillaris are round *in vivo*, and then further correcting for anisometric pixel resolution of this OCT, the accuracy of choriocapillaris size assessment with current OCT could be improved.

Three-dimensional high-density OCT volume data allowed us to study the relationship between choroidal thickness and the choroidal sublayer vascular structures. We found that patients with severely thin choroids (0 to 100 microns) had a lower average vessel density in the innermost choroidal layers as compared to thicker choroids (300 to 400 microns), in whom vessel density was similar between the layers (Graph S1). This is consistent with previous histopathologic evidence of decreased choroidal vessel density and diameter with decreased choroidal thickness [1], [2], [3], [4], [5]. In contrast, previous histopathologic reports did not compare vessel density or size in the different choroidal layers. Our data suggests that with choroidal thinning, vessel density decreases most in the inner layer of the choroid, with less effect on the outer vasculature. The proximity of the choriocapillaris to the RPE allows a high oxygen supply to the highly metabolic outer retina, and the effect of decreased density of choriocapillaris in the setting of early AMD can have important implications on disease progression.

Comparing choroidal thickness among the patient groups, we found RPD eyes had thinner choroids compared to those with AMD without RPD, though these differences were not statistically significant after adjusting for age and gender. Previous studies found that the choroid becomes thinner with increasing age, regardless of disease status [11], [12], [13], [14], [15], [16]. Further analysis of our results demonstrated that, while overall choroidal thickness decreases in both men and women with age, choroid tends to be relatively thinner in younger women and older men (Graph S2). A recent cross-sectional study of young healthy subjects similarly found that women had thinner choroids compared to men for the same axial length [21]. Although the underlying reason for this difference is unclear, future population-based studies are indicated to further explore gender differences in the choroid. Our results suggest that age and gender may play a more important role in determining choroidal thickness than the underlying disease processes.

Limitations of the present study include the use of arbitrary cut-offs for distinguishing between vessel and stroma, compared to histopathology where more specific vascular labeling is possible. However, the pixel intensity cut-offs were standardized and applied to all groups allowing these comparisons. Furthermore, whereas histopathology can differentiate between healthy and ghost choriocapillaris, structural imaging cannot reveal these distinctions. Imaging approaches that are currently in development, such as phase-resolved OCT [22], may allow visualization of choroidal vascular flow to answer this question in the future. Finally, our study selected the layer immediately external to the RPE in patients who did not show evidence of choriocapillaris with the assumption that this layer would be analogous to the choriocapillaris, though this is likely not the case given the qualitative differences noted on our comparisons of sections obtained from each of the groups. Finally, our groups were not balanced with regards to age and gender, but we have controlled for these differences in our statistical analyses to avoid any bias.

In summary, this study is a first step towards using SD-OCT to quantify choroidal vasculature in the aging population *in vivo*. We found distinct differences in the macular choroid between the three groups studied, which were in agreement to previous histopathologic findings. Larger populations, longitudinal studies and the use of phase-resolved approaches will help elucidate the particular sequence of RPE and choroidal changes in the pathogenesis of various AMD subtypes. Further studies are needed to examine these findings in a larger population and to explore whether choriocapillaris atrophy topographically correlates with the location of RPD lesions.

## Supporting Information

Graph S1 **Vessel Density by Choroidal Layer and Average Layer Thickness.** On average, when comparing vessel density and choroidal thickness in each layer for all patients, vessel density is highest in the outermost choroidal layer.
(TIF)

Graph S2 **Total Choroidal Thickness by Age and Gender.** On average, men have thicker choroids in the younger age range than women, but this difference reverses with advanced age (p-value = 0.97).
(TIF)
