# Supplementary figures and images for: A Pilot Study of Morphometric Analysis of Choroidal Vasculature In Vivo, Using En Face Optical Coherence Tomography
Source: PLoS One. 2012 Nov 26;7(11):e48631. doi: 10.1371/journal.pone.0048631 (PMC3506620; doi:10.1371/journal.pone.0048631)

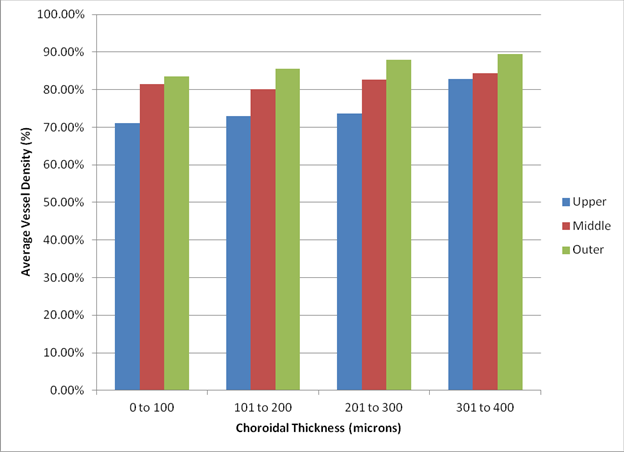

Supplement: Graph S1 — Vessel Density by Choroidal Layer and Average Layer Thickness. On average, when comparing vessel density and choroidal thickness in each layer for all patients, vessel density is highest in the outermost choroidal layer. (TIF) [file pone.0048631.s001.tif]

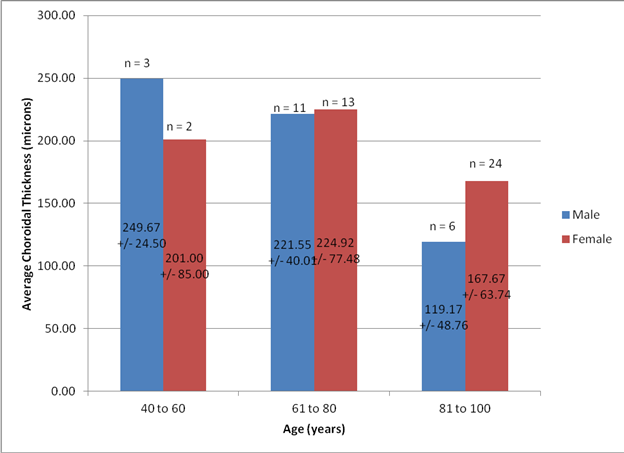

Supplement: Graph S2 — Total Choroidal Thickness by Age and Gender. On average, men have thicker choroids in the younger age range than women, but this difference reverses with advanced age (p-value = 0.97). (TIF) [file pone.0048631.s002.tif]
